# Case report: Identification of a frameshift mutation in GC enrichment and the GCC repeat region of the androgen insensitivity receptor (AR) gene in a patient with complete androgen insensitivity syndrome by whole-exome sequencing (WES) combined with specific PCR and deep sequencing

**DOI:** 10.3389/fgene.2022.1038997

**Published:** 2022-11-25

**Authors:** Xiaojing He, Qingya Ma, Qiaoli Zhang, Xutao Hong, Ming Qi, Yongkai Li, Xiaodong Li

**Affiliations:** ^1^ Department of Obstetrics and Gynaecology, The Second Hospital of Hebei Medical University, Shijiazhuang, China; ^2^ Department of Gynecology, The First Hospital of Hebei Medical University, Shijiazhuang, China; ^3^ Department of Human Reproductive Medicine, Beijing Obstetrics and Gynecology Hospital, Capital Medical University, Beijing, China; ^4^ Beijing Maternal and Child Health Care Hospital, Beijing, China; ^5^ Zhejiang University Medical School Affiliated SRRS Hospital, Zhejiang, China; ^6^ Dian Diagnostics GroupCo., Ltd., Zhejiang, China; ^7^ University of Rochester Medical Center, Rochester, NY, United States; ^8^ Department of Gynecology, The First Affiliated Hospital of Xinjiang Medical University, Urumqi, China

**Keywords:** complete androgen insensitivity syndrome, androgen receptor, whole-exome sequencing, deep sequencing, reproductive chimerism

## Abstract

**Background:** Androgen insensitivity syndrome (AIS) is an X-linked recessive hereditary disease caused due to a reduced or absent function of the androgen receptor (AR) protein encoded by the AR gene (OMIM-Gene# 313,700). Genetic testing is important in the diagnosis, clinical management, and prevention of AIS (MIM# 300,068). The AR (HGNC: 644) pathogenic variant detection rate ranges from 65% to 95% for patients with complete AIS (CAIS) and 40%–45% for patients with partial androgen insensitivity syndrome (PAIS). Identification of a pathogenic mutation in the AR confirms the diagnosis of AIS, especially in the milder forms that may have a phenotypic overlap with other disorders of sex development. Improvement of the molecular diagnostic rate of AIS is urgently required in clinical practice. We reported the results of the molecular diagnosis of a patient with CAIS who failed previously in either the traditional Sanger sequencing or next-generation sequencing (NGS). Using whole-exome sequencing (WES) combined with a special polymerase chain reaction (PCR) and deep sequencing, we successfully identified a pathogenic variant, a hemizygous mutation (c.1395-1396insGA), in the GC-enriched and unstable GCC repeat regions of the AR gene of the proband.

**Conclusion:** The results may be advantageous for the improvement of the detection rate of AIS, as well as other inherited disorders whose disease-causing genes contain GC-enriched and unstable GCC repeat regions.

## Introduction

Androgen insensitivity syndrome (AIS) is the most common cause of 46, XY disorders of sex development, ranging from mild AIS (MAIS) and partial AIS (PAIS) to complete AIS (CAIS) forms of androgen resistance (Liu et al., 2022). CAIS is characterized by a female phenotype in a genetically male (46, XY) individual, whereas PAIS ranges from a predominantly female to predominantly male phenotype, and MAIS patients have normal external male genitalia, while they may suffer from infertility due to defective spermatogenesis (Liu et al., 2022; Zhou et al., 2022). Mutations of variable severity in the AR, the X-linked gene encoding the androgen receptor, cause different forms of AIS, with the total number of public variants of 1,637, unique public DNA variants of 705, individuals with public variants of 1,860, and hidden variants of 47. The establishment of this database was supported by the European Community’s Seventh Framework Programme (FP7/2007-2013). The data were updated on 26 September 2022 (https://databases.lovd.nl/shared/genes/AR). About 65–95% of CAIS patients were found to have an AR mutation (Ono et al., 2018). The identification of a pathogenic mutation in the AR confirms the diagnosis of AIS, especially in the milder forms, which have a phenotypic overlap with other disorders of sex development. Improvement of the molecular diagnostic rate of AIS is essential in clinical practice.

The AR gene contains two polymorphic trinucleotide repeat segments that encode polyglutamine and polyglycine tracts in the N-terminal transactivation domain of its protein. Expansion of the polyglutamine tracts from the normal 9–34 repeats to the pathogenic 38–62 repeats causes spinal and bulbar muscular atrophy (SBMA, also known as Kennedy’s disease) (Grigorova et al., 2017). All the three functional regions of the AR gene are likely to be mutated. Most of the mutations occur in the LBD region, with mutations in exons 5 and 7 as the most common mutations, and the majority of the mutations in this functional region may lead to CAIS. Partial deletion, point mutation, nucleotide insertion, or deletion of the AR gene can cause AIS, among which point mutation is the most common phenomenon, and the deletion type only accounts for 5–10% of AIS (Batista et al., 2018; Ono et al., 2018).

In the present study, we reported an interesting case of the identification of a frameshift mutation in the AR GC-enriched and GCC repeat regions of the AR gene in a patient with CAIS by whole-exome sequencing (WES) combined with a specific polymerase chain reaction (PCR) and deep sequencing.

## Case presentation

**FIGURE 1:**
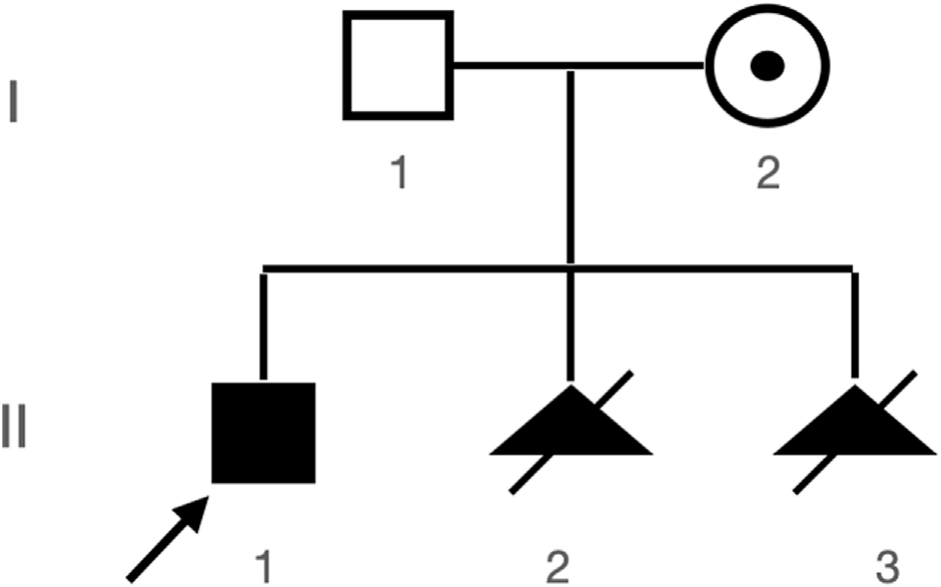
Patient’s pedigree. Proband (II-1) and two subsequent aborted products II-2 and II-3, both with abnormal genitalia seen during prenatal ultrasound and both with 46, XY karyotypes (karyotypes not shown).

**FIGURE 2:**
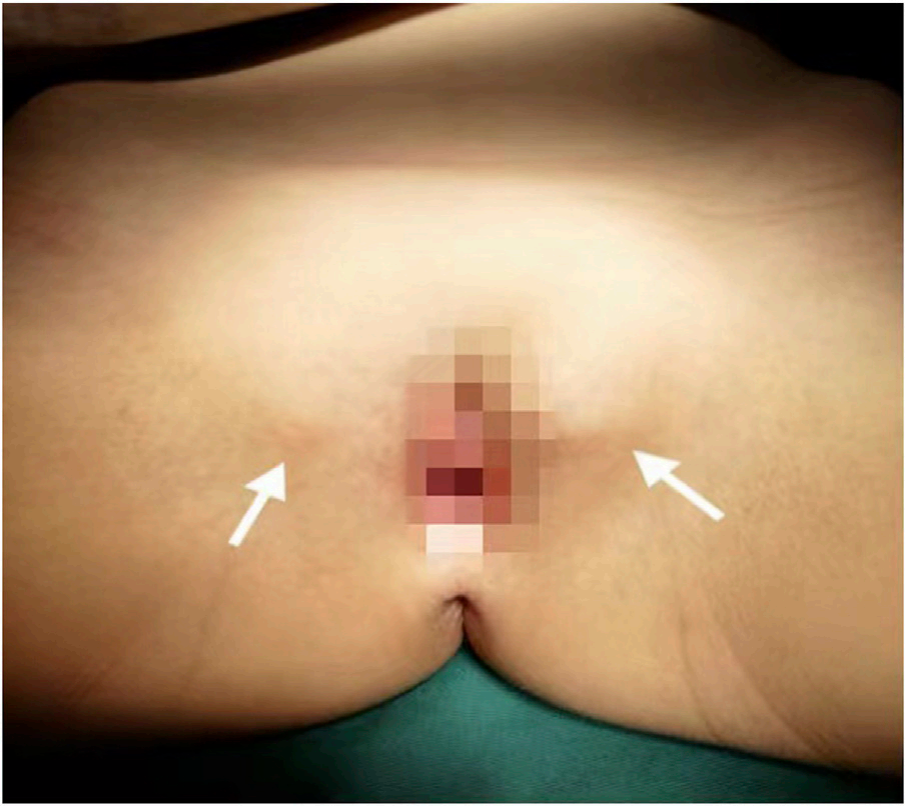
Patient’s female genitalia showing bilateral subcutaneous masses indicated by white arrows.

**FIGURE 3:**
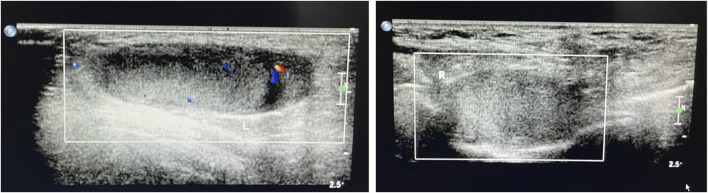
Ultrasound of the bilateral subcutaneous masses in the genital area consistent with testes.

**FIGURE 4:**
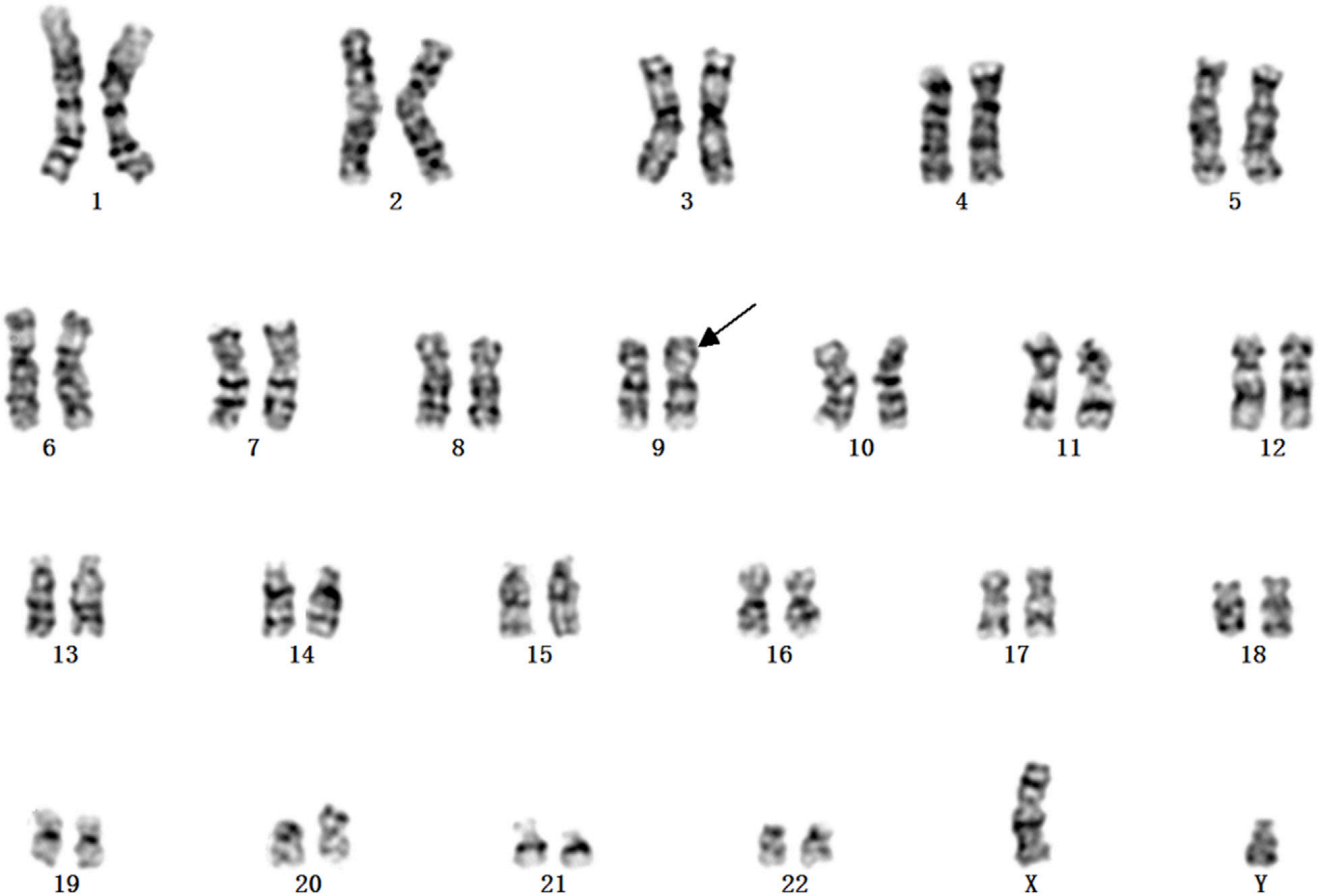
Karyotype of 46, XY in v (9), (p11q13).

**FIGURE 5:**
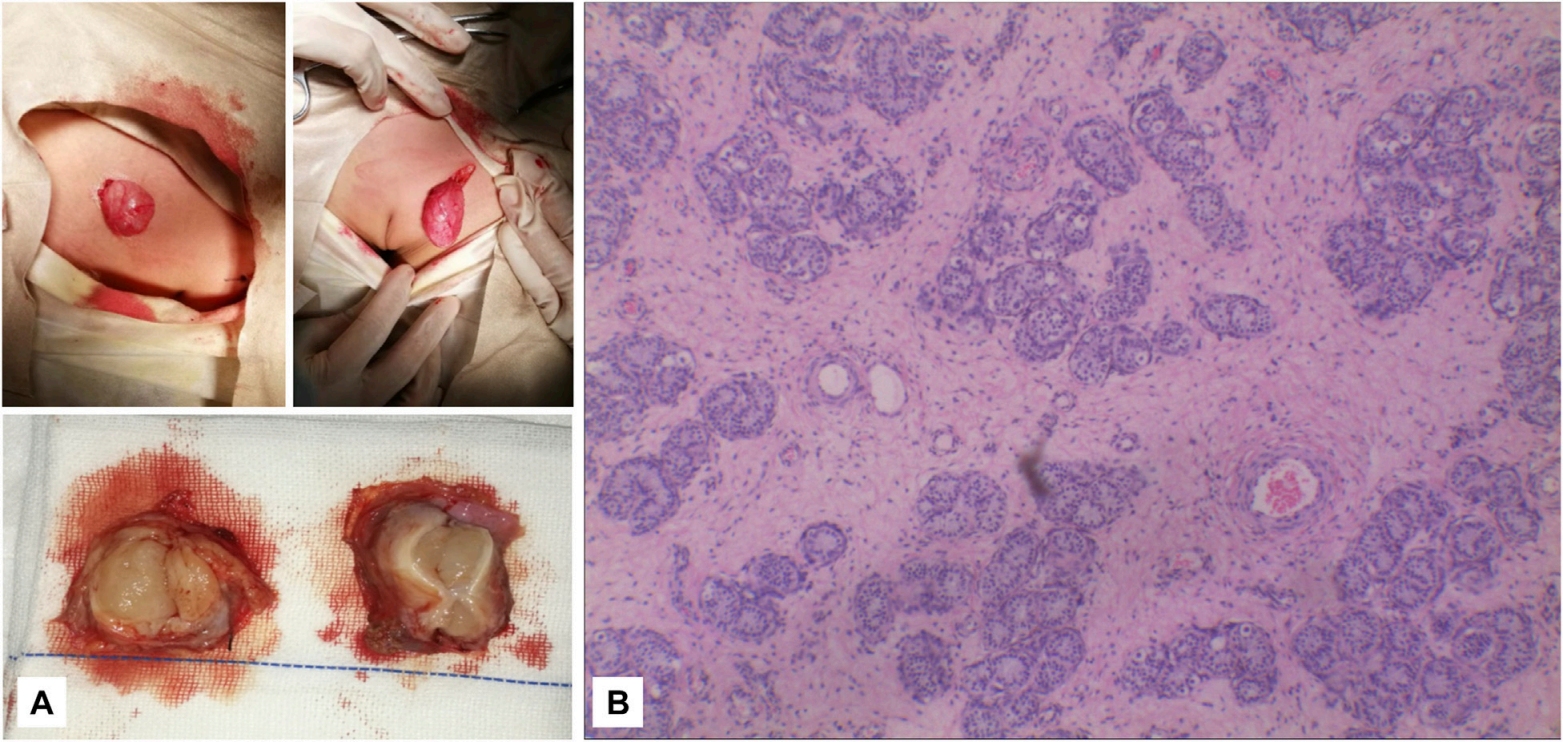
**(A)** It can be seen that the subcutaneous elliptical soft tissue of the bilateral labia majora is like a gonad, and the section is fish-like. **(B)** Pathology: testicular tissue and vas deferens on the left and testicular tissue on the right (magnification, ×2010).

A Chinese family with an X-linked recessive form of 46, XY disorders of sex development (DSD) was admitted to the Clinic of the Second Hospital of Hebei Medical University (Shijiazhuang, China; Figure 1). The proband (II-1) was a 9-year-old phenotypic girl who had not had a menstrual period since childhood at the time of her visit, which could be mainly due to the identification of a bilateral labia subcutaneous mass at the age of 7 years. Physical examination revealed the following findings: without the development of both breasts and armpit hair, girlish vulva, and without pubic hair distribution. The bilateral labia majora might touch a bump of approximately 3 × 2 × 1 cm^3^ in size, which was soft and could be pushed (Figure 2). A mass was found during a 2-year-old inguinal hernia repair, while it was not treated. At the age of 9 years, ultrasound revealed a missing uterus with a subcutaneous mass on both sides of the labia majora resembling an echo of the gonads (Figure 3). Karyotype: 46 XY, V (9), (p11q13) (Figure 4). Sex hormones: follicle-stimulating hormone (FSH): 4.61 mIU/ml, luteinizing hormone (LH): 0.43 mIU/ml, estradiol (E_2_): 4.00 pg/ml, progesterone (p): 0.11 ng/ml, testosterone (T): 0.54 ng/ml, and serum prolactin (PRL): 4.23 ng/ml. Bilateral gonadectomy was performed (Figure 5A). Pathology confirmed that the mass was in the testicular tissue (Figure 5B). The definitive diagnosis was CAIS. Genetic tests on the proband and her parents did not reveal any abnormality. The proband’s mother later had two pregnancies (Figure 1II2II3), and the fetus was diagnosed with AIS by amniocentesis and choriocentesis at 20 and 16 weeks of pregnancy, respectively. The karyotype of the fetus was 46, XY, and ultrasound showed that the external genitalia of the fetus were female. The two fetuses were eventually induced into labor. Prenatal diagnosis of the two adverse pregnancies was confirmed. All exons of the AR gene were sequenced for the amniotic fluid (II4) of the first pregnancy by a well-known molecular diagnostic laboratory in China. However, no pathogenic gene mutations were found. For the second pregnancy, the same test was performed at another better-known molecular diagnostic laboratory. They still did not detect any disease-causing mutation.

In order to find out the causes of multiple bad pregnancies in the mother, WES of the AR gene for the proband was carried out by the PCR and Sanger sequencing, and this experiment was performed in a prenatal diagnosis hospital of Hunan province in China (the name of hospital was not mentioned here); however, a negative result was achieved. The results of Sanger sequencing were checked, in which between the 1,370 and 1,410 bp of exon 1, the stray peaks began to appear and the structure of the PCR product was no longer singular. Then, we continued to perform WES with next-generation sequencing (NGS). As the two abortions of pregnancy were not carried out in another hospital, no fetal tissue sample was available for our testing. The experiment was performed in Genergy Biotechnology Co., Ltd. (Shanghai, China).

## Materials and methods

### DNA extraction and targeted exome sequencing

Samples were collected with written informed consent, in which the study was approved by an Institutional Review Board, and the study was performed in accordance with the principles of the Declaration of the Helsinki. Genomic DNA was extracted from whole-blood samples obtained from the proband and her mother. WES was performed for the proband on exon targets isolated using the Twist Human Core Exome Enrichment system (Twist Bioscience, South San Francisco, CA, United States), and sequence capture, enrichment, and elution were performed, according to manufacturer’s instructions (Twist Bioscience), without modification except for library preparation performed using the NEBNext Ultra II kit (New England Biolabs, Ipswich, MA, United States). For library preparation, 150 ng of each genomic DNA was fragmented by sonication and purified to yield fragments of 150–200 bp. Paired-end adaptor oligonucleotides from the NEB kit were ligated on repaired, a-tailed fragments and were then purified and enriched by 10 PCR cycles. Next, 500 ng of the purified libraries was hybridized using the Twist oligo probe capture library for 16 h in a singleplex reaction. After hybridization, washing, and elution, the eluted fraction was PCR-amplified with eight cycles, and purified and quantified by Qubit and qPCR, in order to obtain a sufficient DNA template for downstream applications. The eluted-enriched DNA sample was then sequenced on an Illumina NovaSeq system (Illumina Inc., San Diego, CA, United States) as 150-bp paired-end reads. Image analysis and base calling were performed by Illumina Real-Time Analysis software with default parameters. At least, 10 G raw data were obtained, with an exome depth of 100X.

### Exome analysis

Sequence reads were mapped to the human genome build (hg19) using the Burrows–Wheeler Aligner tool. The duplicate reads were removed. Then, GATK and SAMtools were applied to create a VCF file containing all the sites with potential variants. The VCF file was filtered based on the two criteria: depth of coverage and Fred score quality (DP > 4 -'QUAL >25). After calling the list of variants, the ANNOVAR software was used to annotate screened variations and connect the three annotation modes, according to the type of gene, region, and filter. However, no pathogenic variant or variant of uncertain significance (VUS) was identified.

### Manual check and reanalysis

**FIGURE 6:**
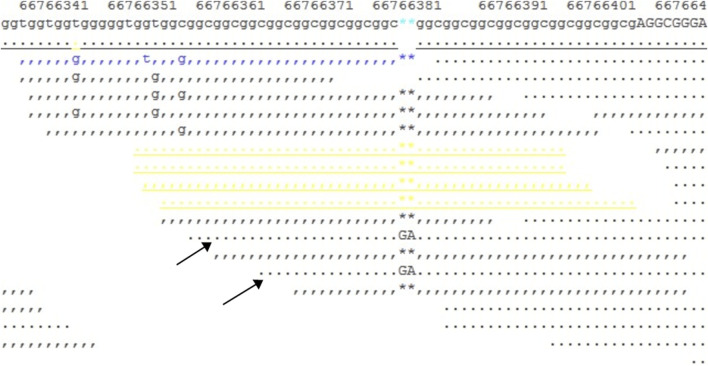
Snapshot of the SAMtools tview command for the BAM file. It shows a 10X coverage in the target region, and two reads (indicated by arrows) show a GA insertion in the GCC repeat region.

We downloaded the original FASTQ and BAM files, then manually checked the original BAM file with the SAMtools command, and found a heterogeneous 2-bp insertion in the GCC repeat region (Figure 6); however, only two reads contained this insertion mutation, with a total of 10X coverage for this region. Then, we reset the filter, with DP ≥ 2, regenerated the VCF file, and finally found a heterogeneous frameshift mutation (c.1395_1396insGA:p.G465fs) in exon 1 of the AR gene.

### Special PCR and Sanger sequencing

Sanger sequencing was used to confirm the results (AR: c.1395_1396insGA: p.G465fs) and to indicate whether the mutation is inherited from the proband’s mother. Primers for the target region of the AR variants were designed by Primer 3.0 online software (http://primer3.ut.ee/) based on the human genome reference sequence and synthesized by Sangon Biotech Co., Ltd. (Shanghai, China). A total of five pairs of primers were designed and synthesized to amplify the DNA template; however, only one works.

Forward primer: ACA​CTC​TCT​TCA​CAG​CCG​AA.

Reverse primer: CAA​GTG​GGA​CTG​GGA​TAG​GG.

Afterward, genomic DNAs of both the proband and her mother were amplified with primer pairs, and the PCR products were then sequenced by Sangon Biotech Co., Ltd. The data were analyzed by Chromas 2.6.6 software.

### Deep sequencing

The target mutation (AR: c.1395_1396insGA) for both gDNA of the proband’s blood (marked sample A), mother’s blood (marked sample B), and mother’s saliva (marked sample C) was amplified using a specially designed primer, which contained a target-specific sequence at the 3′-end, and a sequence that is commonly used in subsequent clonal amplification and sequencing reactions at the 5′-end.

First forward primer:

ACA​CTC​TTT​CCC​TAC​ACG​ACG​CTC​TTC​CGA​TCT -ACA​CTC​TCT​TCA​CAG​CCG​AA.

First reverse primer:

GTG​ACT​GGA​GTT​CCT​TGG​CAC​CCG​AGA​ATT​CCA- CAA​GTG​GGA​CTG​GGA​TAG​GG.

For each sample, using the aforementioned primers, PCR was performed on 10–25 ng of genomic DNA *via* the High-Fidelity PCR system (NEB) in standard thermocycling conditions on a PTC-200 thermocycler. The amplified PCR product was purified using Ampure XP beads. Subsequently, low-cycle amplification was carried out to add the Illumina sequence adapters and indices.

Nested forward primer:

AAT​GAT​ACG​GCG​ACC​ACC​GAA​CAC [NNNNNNNN]ACACTCTTTCCCTACACGACGCTCTTCCGATCT.

Nested reverse primer:

CAA​GCA​GAA​GAC​GGC​ATA​CGA​GAT [NNNNNNNN]GTGACTGGAGTTCCTTGGCACCCGAGAATTCCA.

[NNNNNNNN] represents 8-bp indices.

Afterward, these three amplicons were purified and quantified and were subsequently normalized and pooled. The pooled library was sequenced by MiSeq, using a PE300bp read kit (Illumina Inc.). All samples were required to pass a quality control step of 10000X coverage. Then, FASTQ files were analyzed using a Python-based script.

## Results

For the NGS sample, 11.7 Gbp of raw data were obtained by WES, with a mean coverage of 141-fold. A negative result was obtained under the routine analysis and the filter parameters. We manually checked the BAM files, then reset the filter parameters, and after reanalysis, a heterogeneous frameshift mutation in the AR gene (exon1:c.1395_1396insGA:p.G465fs) was finally found by filtering synonymous SNVs and 1000G (freq >0.01). This mutation leads to a frameshift and generates a premature termination codon. It is a pathogenic mutation according to the American College of Medical Genetics and Genomics (ACMG) standards. However, this mutation was of 2X coverage and was marked as LOW QUAL.

**FIGURE 7:**
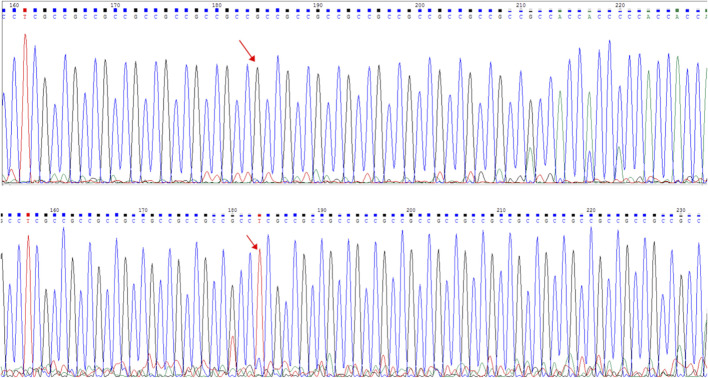
Capillary electrophoresis electropherogram of the AR gene revealed the existence of different mutations (red arrow): C.1395_1396insGA:p.G465fs. Sanger sequencing peak map. The electropherogram at the top corresponds to the mother (I-2), while the bottom electropherogram corresponds to the proband (II-1).

**FIGURE 8:**
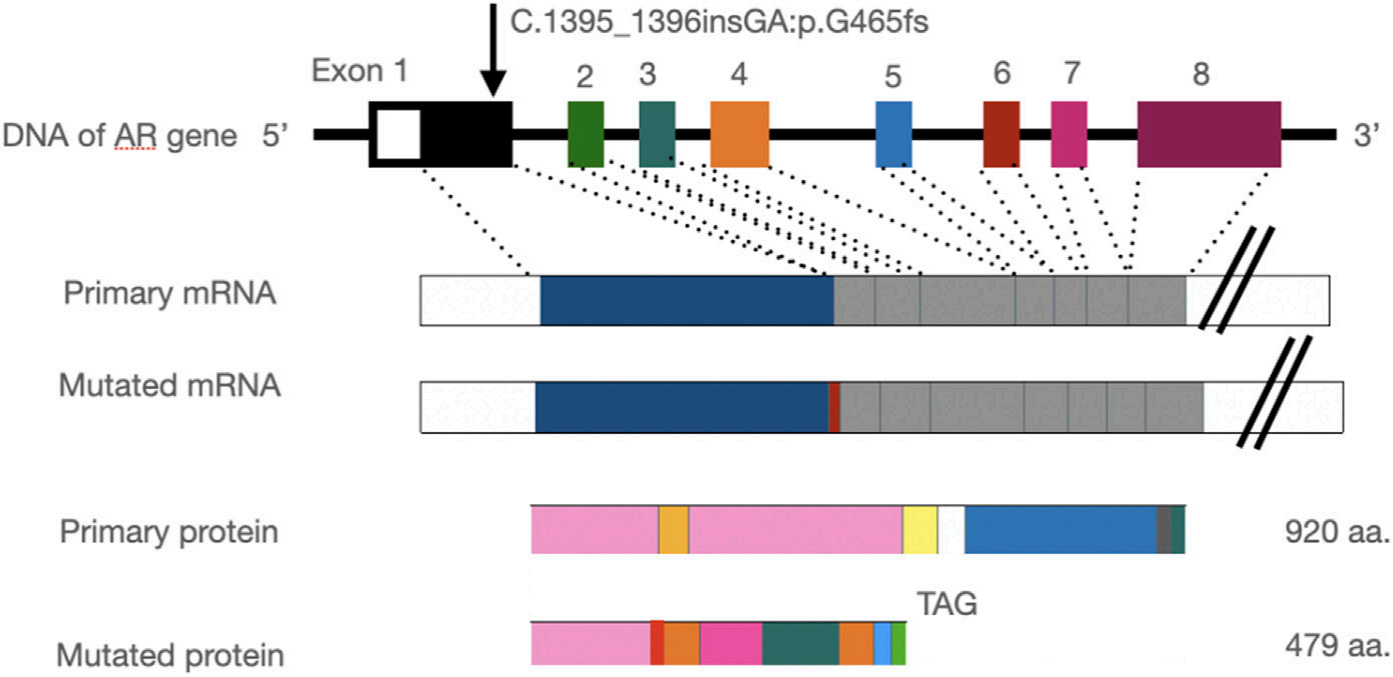
Insertion of the GA base leads to a frameshift mutation in the AR gene and stop codon mutation at codon 479 (TAG), resulting in a truncated protein.

The frameshift mutation was confirmed by Sanger sequencing of the entire coding region using a specific PCR. A total of more than five pairs of primers were designed and tested; however, only one works; furthermore, only the reverse Sanger sequencing results could be distinguished. The results showed that the proband had a hemizygous mutation (c.1395_1396insGA) (Figure 7). The insertion of GA occurred between the eighth and ninth GCC repeats (Figure 6) and caused the frameshift mutation, resulting in the truncation of the AR protein and abolishing its function (Figure 8). However, her mother was negative for this mutation by Sanger sequencing (Figure 7). Furthermore, the results of Sanger sequencing indicated that the number of GCC repeats in the AR gene of the proband was significantly higher than that of her mother. The mother had 15/16 heterozygous repeats, while the proband had about 25 repeats.

Deep sequencing of the mother’s blood revealed that the mother was a mosaic, with a proportion of the mutant less than 30%. In addition, the mother had 15/16/17 heterozygous GGC repeats, while the proband had 24/25/26 repeats.

## Discussion and conclusion

AIS is an X-linked recessive genetic disease. At present, it is widely accepted that AIS is closely correlated with the abnormality of the androgen receptor, which is caused by the mutation of the androgen receptor gene (Poon et al., 2021). Because of the completely feminized phenotype of CAIS, it is difficult to diagnose and treat AIS patients as soon as possible through clinical surgery; thus, genetic testing is particularly important. More than 50% of the relatives of CAIS patients may have the same disease. Genetic testing can confirm a clinical diagnosis, on one hand, and distinguish carriers on the other. When a family has a patient with CAIS, identifying the type of mutation and prenatal diagnosis is the only way to avoid birth and reduce the risk of recurrence. Although different AIS-associated AR mutations have been reported, providing genetic counseling for patients with DSD or couples may remain challenging. Only few cases of AIS with the AR gene mutations have been diagnosed prenatally.

This patient was diagnosed with CAIS when her mother was in the second trimester of pregnancy. In order to avoid the birth of children with AIS, we performed genetic testing in time, while no abnormal mutation was found. Similarly, when the patient’s mother became pregnant for the third time, the results of genetic testing showed no abnormality. Regarding its rarity and being under research, for the importance of early diagnosis and treatment and how to diagnose genetic problems more clearly, we attempted to share our cases.

Patients with the AR mutations may present a genital phenotype ranging from ambiguous genitalia in partial AIS (PAIS) to female genitalia in CAIS (1). In terms of genetic counseling, most of the new mutations in AIS patients are germline mutations; thus, they may originate from a single germ cell or germ cell chimerism. In 70% of the cases, mutations are germline mutations and are transmitted in an X-linked manner through the carrier mothers. In about 30% of the cases, mutations appear *de novo* in the patient. *De novo* mutations may originate from the mother either in a single germ cell or as a germ cell mosaicism and then, present as hemizygous germline mutations in the 46, XY offspring (Farah et al., 2021). Because the mother transmitted the mutation twice, germ cell mosaicism could be assumed, while the germ cells of the mother could not be profoundly studied.

The AR gene is located on the long arm of the X chromosome (Xq11∼12). The AR contains four domains: (I) the amino terminal activation domain (NTD), (II) the DNA-binding domain (DBD), (III) the hinge region (HR), and (IV) the carboxyl ligand-binding domain (LBD) (4). AR exon 1 encodes the entire N-terminal domain (NTD) (a.a. 1–556), which comprises the bulk of the AR and is the least conserved of the four domains. To date, more than 500 mutations in the AR have been described, including point mutations, frameshift mutations leading to premature termination of transcription, and gross deletions, as well as small deletions or insertions scattered around the entire sequence of the gene (Gottlieb et al., 2004; Birgit et al., 2005). Mutations in the AR gene are mostly missense and enriched in the LBD of protein (Ono et al., 2018), and about 65–95% of CAIS patients were found to have a mutation in the AR gene because some deep intronic mutations would be missed by the WES method (Känsäkoski et al., 2016).

In the present study, we found some mutations in exon regions that were missed by WES. The exon 1 of the AR gene, the CDS from 1,370 to 1,420, is a GC-enriched region, which contains five GGT repeats and 15 GGC repeats, coding 25% glycine, and this region is hard to be disrupted in the fragment steps of the WES library construction. Thus, this region is always associated with a low coverage in WES, resulting in a negative result. Second, the number of GGT or GGC repeats may increase, expanding as the gene is passed from the mother to the child, and the PCR steps may also cause the number of repeats to be unstable. In the present study, we found that the number of proband’s GGC repeats was from 25 to 27; one side of the frameshift mutation included 15–16 GGC repeats and caused a terrible mismapping (Figure 6), and these unstable repeats might also cause great difficulty in the determination of the results of Sanger sequencing, in which the GGC repeats may mislead to a frameshift mutation. We tested several pairs of PCR primes, and the stray peaks always appeared to disturb us to manually identify this frameshift mutation in Sanger sequencing. In addition, the low chimerical rate may also lead to a negative result of Sanger sequencing (Figure 7). To eliminate these deficiencies, we used WES combined with special PCR and deep sequencing, in order to identify a hemisphere pathogenic frameshift mutation in the proband, while her mother was a carrier with chimerical reproduction. Furthermore, we also found that the GGC repeats were 15/16 chimeric in the mother, while there were 25/26 repeats in the proband; however, no study has reported whether the increase in the number of GCC repeats is correlated with the incidence of the disease. These results perfectly explained the proband and the two abnormal fetuses of her mother. The proband and her mother will both benefit from these results, and the mother should pay attention to prenatal diagnosis or the preimplantation genetic test (PGT-M) in the next pregnancy. However, we did not achieve the specimens of the induced labor, and hence, it could not be further verified.

In this study, when the AR gene mutation was passed on to the next generation, the repetition number of the offspring increased and GA insertion was introduced, resulting in a frameshift mutation. The insertion or deletion of a single nucleotide or a pair of nucleotides can lead to a reading frame frameshift mutation, which often leads to protein truncation. The purpose of Deping NGS is to verify that the mother is Sanger-negative and speculates on possible reproductive chimerism (rather than new mutations). The process of finding the cause of the disease is difficult and bumpy, but this result explains the cause of the disease in the family. More specific genetic counseling is provided for mothers who are carriers. It has a clear guiding significance for the second pregnancy. Efficient and accurate gene detection can detect families with abnormal sexual development and their genetic carriers as early as possible. In this case, the family genetic analysis of AIS and prenatal diagnosis of a high-risk fetus, combined with clinical data to indicate whether the fetus is sick or not, are also of great significance to avoid the recurrence of AIS in families (Birgit et al., 2005). When you highly doubt the possibility of missed diagnosis in genetic testing, you should boldly make a decision to retest and reanalyze. We successfully identified a pathogenic variant, a hemizygous mutation (c.1395-1396insGA), in the GC-rich and GCC repeat region of the AR gene of the proband by WES combined with the specific PCR and deep sequencing, and her mother was a carrier with a germline mosaic. Because this mutation is very special, with a high GC content, and it is a GCC repeat region, it is difficult to accurately identify it by Sanger sequencing. We conjectured the same frameshift mutation that would be verified in these two induced products with the same clinical features. Therefore, if the result of Sanger sequencing is negative, it should be retested with NGS. Regrettably, the specimens are unavailable to us as the procedures were performed at another hospital.

In conclusion, a case of AIS was diagnosed, while high-throughput NGS or Sanger sequencing did not find pathogenic mutations, which should be analyzed manually, especially mutations that are hidden in repeat regions, such as GCC. Manual analysis can compensate for the deficiency of high-throughput NGS. For cases of suspected reproductive chimerism or cases where pathogenic mutations in mothers cannot be found by Sanger sequencing or high-throughput NGS, targeted, high-depth, NGS can be considered for verification.

## Data Availability

The data presented in the study are deposited in the clinvar repository, https://submit,ncbi,nlm,nih,gov/subs/variationclinvar/SUB12287204.

## Nomenclature

AIS: androgen insensitivity syndrome
WES: whole-exome sequencing
NGS: next-generation sequencing
